# Oteseconazole versus fluconazole for the treatment of severe vulvovaginal candidiasis: a multicenter, randomized, double-blinded, phase 3 trial

**DOI:** 10.1128/aac.00778-23

**Published:** 2023-12-14

**Authors:** Xiaoqian Wang, Lihong Chen, Hongjie Ruan, Zhengai Xiong, Wenying Wang, Jin Qiu, Weihua Song, Chunlian Zhang, Fengxia Xue, Tianhua Qin, Bei Zhang, Ruifang An, Xiping Luo, Wei Wang, Songling Zhang, Yunlang Cai, Jiali Kang, Henan Deng, Shangrong Fan, Manhua Cui, Shijin Wang, Xiaowan Luo, Zhiying Su, Jing Shu, Quanren Wang, Fang Wang, Jianling Bai, Qinping Liao

**Affiliations:** 1 Department of Obstetrics and Gynecology, Beijing Tsinghua Changgung Hospital, Tsinghua University School of Clinical Medicine, Beijing, China; 2 Department of Gynecology, Shaanxi Provincial People’s Hospital, Shaanxi, China; 3 Department of Gynecology, Women’s Hospital of Nanjing Medical University, Nanjing Maternity and Child Health Care Hospital, Jiangsu, China; 4 Department of Obstetrics and Gynecology, The Second Affiliated Hospital of Chongqing Medical University, Chongqing, China; 5 Department of Gynecology, The First Affiliated Hospital of Xi’an Medical University, Shaanxi, China; 6 Department of Obstetrics and Gynecology, Shanghai Tong Ren Hospital, Shanghai Jiaotong University School of Medicine, Shanghai, China; 7 Department of Gynecology, Obstetrics Women & Children’s Health Care Hospital of Linyi, Shandong, China; 8 Department of Gynecology, Taihe Hospital Affiliated Hospital of Hubei University of Medicine, Hubei, China; 9 Department of Gynecology, Tianjin Medical University General Hospital, Tianjin, China; 10 Department of Gynecology, Urumqi Maternal and Child Health Hospital of Xinjiang Uygur Autonomous Region, Xinjiang, China; 11 Department of Gynecology, Xuzhou Central Hospital, Jiangsu, China; 12 Department of Obstetrics and Gynecology, The First Affiliated Hospital of Xi’an Jiaotong University, Shaanxi, China; 13 Department of Gynecology, Guangdong Women and Children Hospital, Guangdong, China; 14 Department of Obstetrics and Gynecology, Peking University Third Hospital, Beijing, China; 15 Department of Obstetrics and Gynecology, The First Hospital of Jilin University, Jilin, China; 16 Department of Obstetrics and Gynecology, Zhongda Hospital Southeast University, Jiangsu, China; 17 Department of Gynecology, Guangzhou First People’s Hospital, Guangdong, China; 18 Department of Gynecology, The First People’s Hospital of Chenzhou, Hunan, China; 19 Department of Obstetrics and Gynecology, Peking University Shenzhen Hospital, Guangdong, China; 20 Department of Obstetrics and Gynecology, The Second Hospital of Jilin University, Jilin, China; 21 Department of Gynecology, The First Affiliated Hospital of Xinxiang Medical University, Henan, China; 22 Department of Gynecology, Boai Hospital of Zhongshan, Guangdong, China; 23 Department of Gynecology, Women and Children’s Hospital of Xiamen University, Fujian, China; 24 Department of Reproductive Endocrinology and Gynecology, Zhejiang Provincial People’s Hospital, Zhejiang, China; 25 Clinical Research and Development, Jiangsu Hengrui Pharmaceuticals Co., Ltd., Shanghai, China; 26 Department of Biostatistics, School of Public Health, Nanjing Medical University, Jiangsu, China; University of Iowa, Iowa City, Iowa, USA

**Keywords:** oteseconazole, *Candida albicans*, severe vulvovaginal candidiasis, fluconazole, *Candida glabrata*

## Abstract

Vulvovaginal candidiasis (VVC) is a common condition among women. Fluconazole remains the dominant treatment option for VVC. Oteseconazole is a highly selective inhibitor of fungal CYP51. This randomized, double-blinded, phase 3 trial was conducted to evaluate the efficacy and safety of oteseconazole compared with fluconazole in treating severe VVC. Female subjects presenting with vulvovaginal signs and symptoms score of ≥7 and positive *Candida* infection determined by potassium hydroxide test or Gram staining were randomly assigned to receive oteseconazole (600 mg on D1 and 450 mg on D2) or fluconazole (150 mg on D1 and D4) in a 1:1 ratio. The primary endpoint was the proportion of subjects achieving therapeutic cure [defined as achieving both clinical cure (absence of signs and symptoms of VVC) and mycological cure (negative culture of *Candida* species)] at D28. A total of 322 subjects were randomized and 321 subjects were treated. At D28, a statistically significantly higher proportion of subjects achieved therapeutic cure in the oteseconazole group than in the fluconazole group (66.88% vs 45.91%; *P* = 0.0002). Oteseconazole treatment resulted in an increased proportion of subjects achieving mycological cure (82.50% vs 59.12%; *P* < 0.0001) and clinical cure (71.25% vs 55.97%; *P* = 0.0046) compared with fluconazole. The incidence of treatment-emergent adverse events was similar between the two groups. No subjects discontinued study treatment or withdrew study due to adverse events. Oteseconazole showed statistically significant and clinically meaningful superiority over fluconazole for the treatment of severe VVC and was generally tolerated.

## INTRODUCTION

Vulvovaginal candidiasis (VVC) is a mucosal fungal infectious disease that causes vulvovaginal itching, irritation, burning, soreness, fissuring, redness, vaginal discharge, and dyspareunia (1). Approximately 80% of women experience at least one occurrence of VVC in their lifetime, with a much higher incidence in women of reproductive age than menopausal women, seriously impacting their quality of life (2, 3). Despite that VVC is predominantly caused by *Candida albicans* (*C. albicans*), non-*C*. *albicans* species, especially *Candida glabrata* (*C. glabrata*), have been increasingly recognized as pathogens associated with VVC (4). Oral fluconazole remains the dominant treatment option for VVC and has achieved great success (5, 6). However, concerns have been raised regarding the development of drug resistance and reduced activity against non-*C*. *albicans* species (7, 8). In a previous *in vitro* antifungal susceptibility study of *Candida* species, it was observed that the resistance to fluconazole was noticeably higher in *C. glabrata* compared to *C. albicans*, with resistance rates of 73.3% and 16.6%, respectively (9). In addition, despite the remarkable therapeutic efficacy observed during fluconazole treatment, approximately 50% of the women experience disease relapse within 6 months of fluconazole cessation (7, 8). Therefore, novel antifungal drugs are still in great need.

Oteseconazole (formerly designated as VT-1161) is a novel, oral, highly selective inhibitor of fungal CYP51. Oteseconazole demonstrated a more than 2,000-fold selectivity for fungal CYP51 over human CYPs, thus potentially reducing the safety concerns caused by off-target toxicities (10). In an antifungal activity test against a panel of clinical isolates of *Candida* species, oteseconazole showed superior activity, with an average potency of a more than 40-fold greater than fluconazole for most species (11). More importantly, for fluconazole-resistant *C. glabrata*, the minimal inhibitory concentration (MIC) of oteseconazole was 64-fold lower than fluconazole (11, 12).

Previous phase 2 and phase 3 global studies with oteseconazole have demonstrated clinically meaningful efficacy in treating recurrent vulvovaginal candidiasis (RVVC), which was defined as ≥3 episodes of VVC within ≤12 months (13 – 16). In this phase 3 study, we compared the efficacy and safety of oteseconazole versus fluconazole for the treatment of severe VVC in Chinese women.

## MATERIALS AND METHODS

### Subject population

Female subjects aged ≥18 and ≤75 years with vulvovaginal signs and symptoms (VSS) score ≥7 were enrolled. The VSS score is determined with a standardized and predefined scoring system in which each sign (congestion and edema, scratches, rhagades and erosions, secretion volume) and symptom (itching, pain) was given a numerical rating based on severity. The severity of each item was graded from 0 to 3 (absent = 0, mild = 1, moderate = 2, and severe = 3), except for the item “scratches, rhagades and erosions” (absent = 0 and present = 3) (17). In addition, positive *Candida* species determined by the microscopic examination of a wet mount of vaginal discharge with potassium hydroxide (KOH) or Gram staining were required. For women of child-bearing potential, a negative pregnancy test at screening was a prerequisite, as well as the usage of contraception throughout the study and 6 months after the last administration of treatments. Subjects with RVVC or a history of RVVC or with concomitant vulvovaginitis caused by other pathogens were excluded. Other major exclusion criteria included the use of antifungal treatments (systemic and/or topical), CYP3A4 substrates, CYP3A4 inducers or inhibitors, and vulvovaginal corticosteroids; estrogen replacement therapy within 7 days before randomization; use of systemic corticosteroid treatments within 30 days before randomization or systemic immunosuppressant treatments within 90 days before randomization; a history of cervical cancer; or moderate to severe hepatic and/or renal disorders.

This study was performed between April 2021 and October 2021 at 26 sites in China. The study was conducted in accordance with the Declaration of Helsinki and the Guidelines for Good Clinical Practice. Each study site obtained independent ethics committee approval before the study initiation, and each subject provided written informed consent before participating in the study. This study was registered in ClinicalTrials.gov (NCT04956419).

### Study design and procedures

Subjects were randomly assigned in a 1:1 ratio to the oteseconazole group or the fluconazole group. Subjects, investigators, site personnel, and the sponsor were all blinded to the treatment. To maintain blinding, all randomized subjects received matching oteseconazole or fluconazole placebo based on the treatment regimen, and study treatments had the same appearance.

In the oteseconazole group, subjects were orally administered with oteseconazole 600 mg (150 mg per capsule) and fluconazole matching placebo 150 mg (50 mg per capsule) on D1, oteseconazole 450 mg on D2, and fluconazole matching placebo 150 mg on D4. In the fluconazole group, subjects were orally administered with fluconazole 150 mg and oteseconazole matching placebo 600 mg on D1, oteseconazole matching placebo 450 mg on D2, and fluconazole 150 mg on D4. If clinically meaningful relief was not achieved, symptoms worsened, or signs of intolerance appeared and microscopic examination confirmed positive *Candida* infection 1 week after the initial dose, rescue therapy with clotrimazole vaginal tablet (Canesten, Bayer), 500 mg on D1 and D4, could be provided at the discretion of the investigator. A subject diary was used to record adverse events (AEs), study drug exposure, and concomitant medications from screening till the last visit and was retrieved at each visit.

### Assessments and outcome measures

At baseline, D14, and D28, assessments of efficacy were performed in terms of VVC VSS scores (17); vaginal secretion fungal culture, strain identification, and *in vitro* antifungal susceptibility testing conducted by qualified central laboratory per Clinical and Laboratory Standards Institute M59 and M60 guidelines (18, 19) and European Committee on Antimicrobial Susceptibility Testing methods; and microscopic examination of vaginal secretion pathogen by KOH or Gram staining. The baseline was defined as the last measurement before treatment administration.

Safety was assessed by monitoring AEs, physical examinations, laboratory tests (hematology, clinical chemistry, and urinalysis), vital signs, and electrocardiograms (ECGs) from the time of signed consent to the end of the study. For women with child-bearing potential, a blood pregnancy test was performed at screening and at the D28 visit.

### Endpoints

The primary efficacy endpoint was the proportion of subjects achieving therapeutic cure at D28, defined as the achievement of both clinical cure (absence of signs and symptoms of VVC) and mycological cure (negative culture of vaginal swabs for growth of *Candida* species). The secondary efficacy endpoints included the proportion of subjects reaching therapeutic cure at D14, the proportion of subjects achieving clinical cure at D14 and D28, the proportion of subjects achieving mycological cure at D14 and D28, and the percentage of subjects taking rescue medication during study treatment.

### Statistical analyses

Efficacy analyses were performed in the modified intention-to-treat (mITT) population, which included all randomized subjects with positive mycological culture of *Candida* species at screening. For efficacy analyses, categorical data were summarized as percentages, with 95% confidence intervals (CIs) calculated by the Clopper-Pearson method. Treatment differences between the two groups were detected using Fisher’s exact probability test, and the 95% CI of rate difference was calculated by the normal approximate method. For the endpoints of therapeutic cure, clinical cure, and mycological cure rates, analyses in the mITT subpopulation of subjects testing positive for *C. albicans* were also performed. Safety analysis set included all randomized subjects who received at least one dose of study treatment. AEs were coded using the Medical Dictionary for Regulatory Activities (MedDRA) version 24.1 and summarized by preferred term (PT) with a breakdown of treatment groups. All analyses were conducted using SAS software version 9.4 (SAS Institute Inc, Cary, North Carolina).

A sample size of 320 subjects (1:1 randomization) was required to provide 85% power to detect a non-inferiority margin of 12% between the oteseconazole group and the fluconazole group, with a one-sided α of 0.025 of Fisher’s exact probability test, a 5% negative rate of vaginal swabs culture, a 10% dropout rate, and assuming therapeutic cure rates of 70% and 65% for the oteseconazole and fluconazole groups at D28 visit, respectively (13).

If the lower limit of the 95% CI for the between group difference was greater than −12% in the primary endpoint analysis, non-inferiority test was met. If non-inferiority was demonstrated, oteseconazole was to be tested for superiority compared with fluconazole for the primary endpoint. All statistical tests for the efficacy endpoints, except for the non-inferiority test in the primary analysis, were tested using a two-sided α of 0.05.

## RESULTS

**Fig 1:**
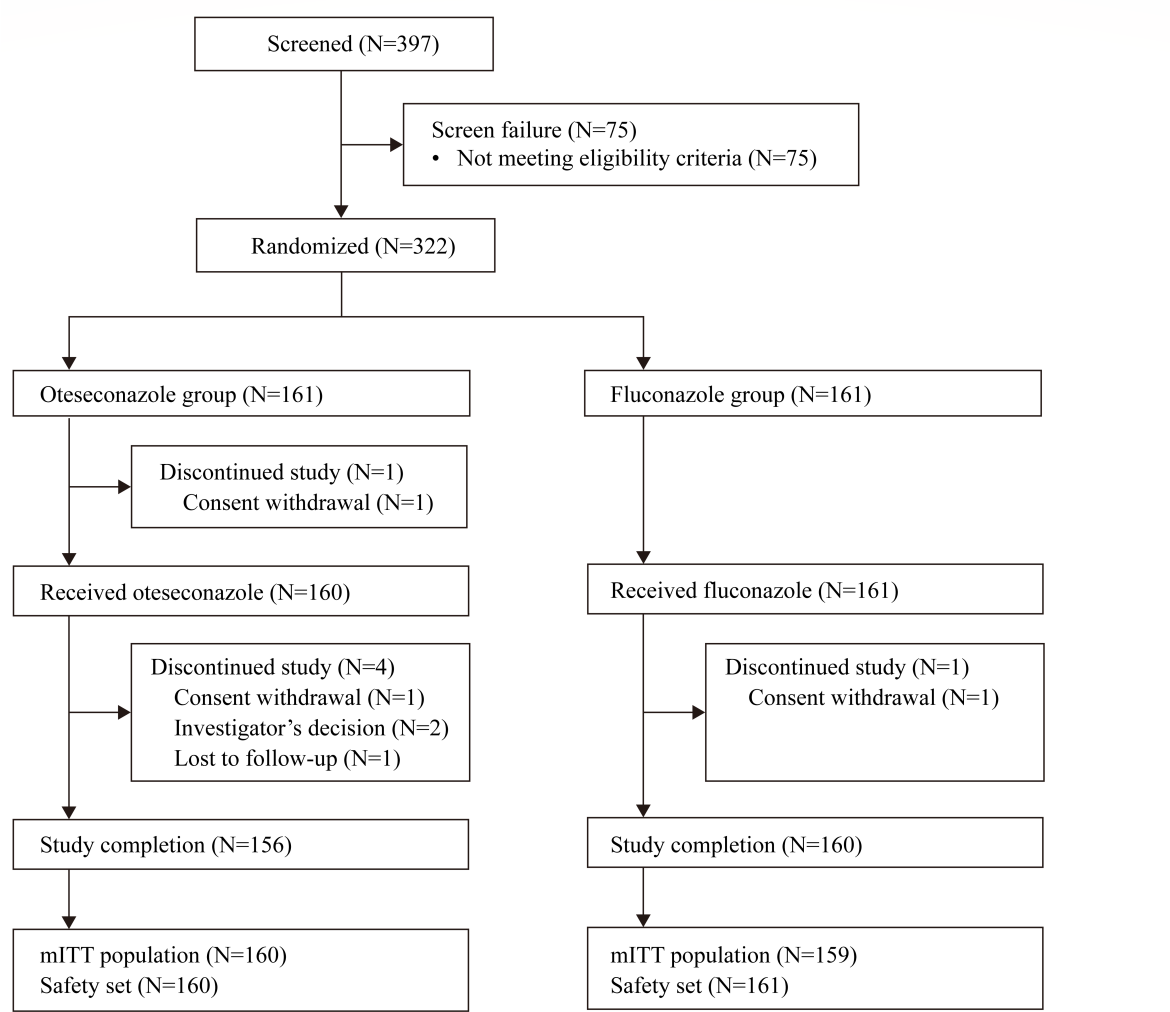
Subject disposition. Abbreviations: N, number of subjects; mITT, modified intention-to-treat.

A total of 322 female subjects were randomized, with 161 subjects each in the oteseconazole and fluconazole groups, respectively. A total of 319 subjects had a confirmed positive culture for *Candida* species at screening and were included in the mITT population for efficacy evaluations. All subjects received treatments and were included in the safety analysis set, except for one subject in the oteseconazole group. A total of 316 subjects completed the study, with the reasons for premature study withdrawal being consent withdrawal (*n* = 3), investigator’s decisions (*n* = 2), and lost to follow-up (*n* = 1) (Fig. 1).

The demographics and baseline disease characteristics were generally balanced between the two groups. Baseline mean VSS score was comparable between the oteseconazole (8.7) and fluconazole groups (8.4), with a range of 7–14 in both groups. Most subjects tested positive culture for *C. albicans* (78.1%), followed by *C. glabrata* (15.4%). *In vitro* susceptibility testing demonstrated that 309 and 233 strains were sensitive to oteseconazole and fluconazole, 6 and 32 strains were resistant to oteseconazole and fluconazole, 0 and 53 strains were dose-dependently sensitive to oteseconazole and fluconazole, respectively (Table 1). Oteseconazole yielded a lower MIC value for 90% of the organisms (MIC_90_) than fluconazole for clinical isolates of *C. albicans* (0.25 vs 4 µg/mL) and *C. glabrata* (4 vs 16 µg/mL) (Table S1).

**TABLE 1:** Demographics and baseline disease characteristics (mITT)^*c*^

	Oteseconazole (*N* = 160)	Fluconazole (*N* = 159)
Age (year)		
Mean (SD)	29.9 (8.0)	31.2 (7.5)
Median (min, max)	29 (18, 70)	31 (18, 50)
Ethnicity		
Han	152 (95.0)	149 (93.7)
Other	8 (5.0)	10 (6.3)
Weight (kg)		
Mean (SD)	56.6 (9.2)	55.6 (8.7)
Median (min, max)	55.0 (42.0, 95.0)	55.0 (40.0, 90.0)
BMI (kg/m^2^)		
Mean (SD)	21.4 (3.5)	21.5 (3.2)
Median (min, max)	20.8 (15.6, 37.1)	20.8 (16.4, 32.9)
Composite VSS score		
Mean (SD)	8.7 (1.8)	8.4 (1.8)
Median (min, max)	8.0 (7, 14)	8.0 (7, 14)
*Candida* species^*a*^		
*Candida albicans*	128 (80.0)	121 (76.1)
*Candida glabrata*	22 (13.8)	27 (17.0)
*Candida tropicalis*	5 (3.1)	3 (1.9)
*Candida krusei*	1 (0.6)	2 (1.3)
*Candida spherical*	2 (1.3)	2 (1.3)
*Candida parapsilosis*	1 (0.6)	2 (1.3)
*Kodamaea ohmeri*	0	1 (0.6)
*Candida dubliniensis*	1 (0.6)	0
*Saccharomyces cerevisiae*	0	1 (0.6)
*Candida lusitaniae*	0	1 (0.6)
Susceptibility testing^*b*^		
Sensitive	309 (96.6)	233 (72.8)
Resistant	6 (1.9)	32 (10.0)
Dose-dependently sensitive	0	53 (16.6)
Wild strain	2 (0.6)	2 (0.6)
Unknown	3 (0.9)	0

^*a*^ Determined by mycological culture of vaginal secretion.

^*b*^ A total of 320 fungal strains were isolated from 319 subjects and 320 was set as the denominator. One subject in the fluconazole group presented with two strains (*Candida parapsilosis* and *Kodamaea ohmeri*).

^*c*^ Data are *n* (%) unless otherwise indicated. Abbreviations: BMI, body mass index; max, maximum; min, minimum; mITT, modified intention-to-treat; *N*, number of subjects in the mITT population; SD, standard deviation; VSS, vulvovaginal signs and symptoms.

**Fig 2:**
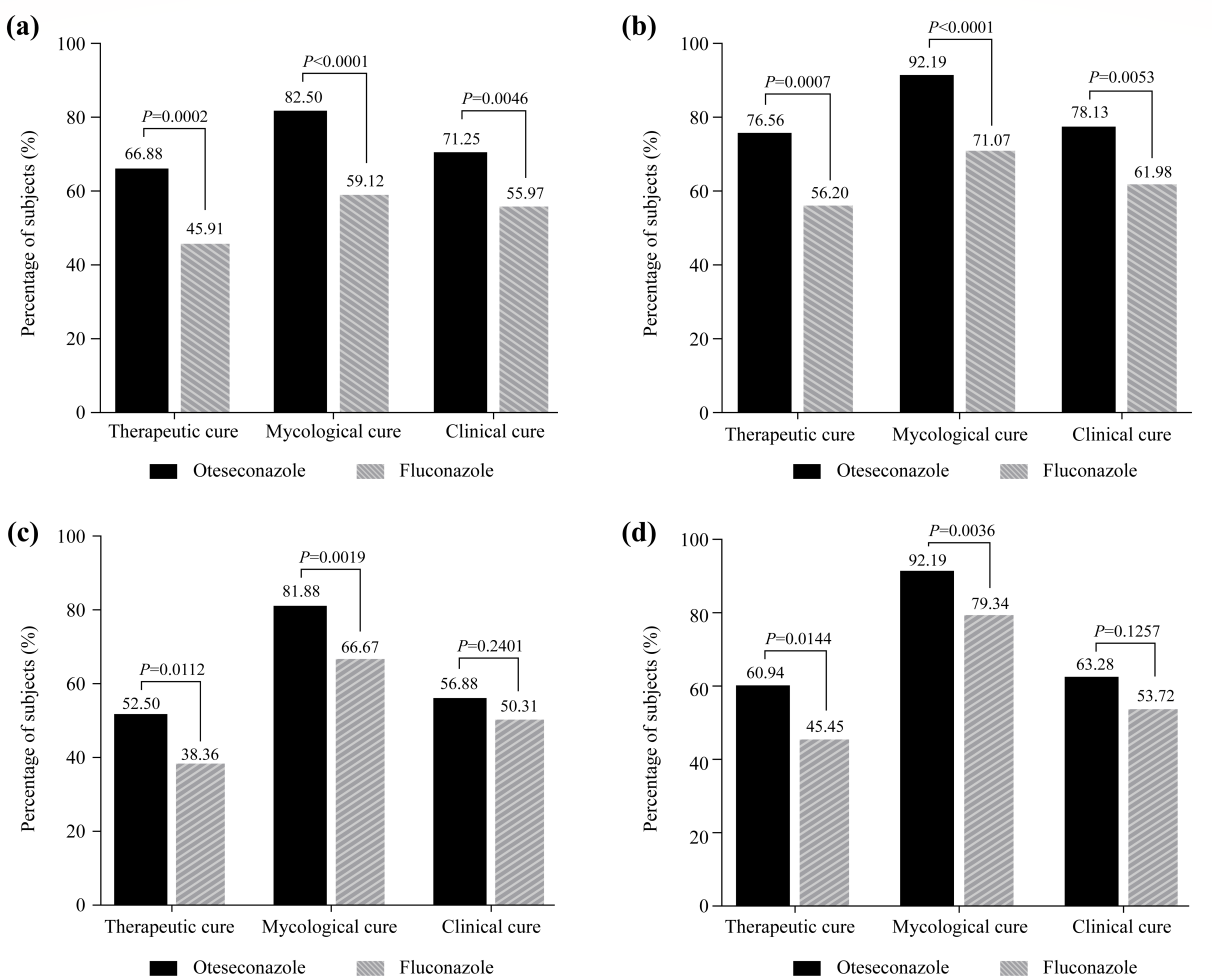
Efficacy results at D28 in the mITT population (**a**), at D28 in the mITT subpopulation with positive culture for *Candida albicans* (**b**), at D14 in the mITT population (**c**),and at D14 in the mITT subpopulation with positive culture for *Candida albicans* (**d**). Abbreviation: mITT, modified intention-to-treat.

At D28, a statistically significantly higher proportion of subjects achieved therapeutic cure in the oteseconazole group than in the fluconazole group [66.88% vs 45.91%; difference (95% CI): 20.96% (10.32, 31.60); *P* = 0.0002] (Fig. 2a; Table S2). Consistent results were observed in subjects with a positive culture of *C. albicans*, where therapeutic cure was reported in 76.56% of subjects in the oteseconazole group versus 56.20% in the fluconazole group at D28 [difference (95% CI): 20.36% (8.87, 31.85); *P* = 0.0007] (Fig. 2b; Table S2).

Results of the secondary endpoints were supportive of the primary endpoint. At D28, the proportion of subjects achieving mycological cure was significantly higher in the oteseconazole group than in the fluconazole group [82.50% vs 59.12%; difference (95% CI): 23.38% (13.73, 33.03); *P* < 0.0001], which was also true in the subjects with a positive culture of *C. albicans* [92.19% vs 71.07%; difference (95% CI): 21.11% (11.79, 30.43); *P* < 0.0001]. A significantly higher clinical cure rate at D28 was shown with the treatment of oteseconazole versus fluconazole, in the overall mITT population [71.25% vs 55.97%; difference (95% CI): 15.28% (4.85, 25.70); *P* = 0.0046], as well as in the subpopulation of subjects with positive *C. albicans* [78.13% vs 61.98%; difference (95% CI): 16.14% (4.91, 27.37); *P* = 0.0053] (Fig. 2a and b; Table S2).

At D14, the proportions of subjects experiencing therapeutic cure and mycological cure were significantly higher in the oteseconazole group than in the fluconazole group [therapeutic cure rate: 52.50% vs 38.36%; difference (95% CI): 14.14% (3.32, 24.95); *P* = 0.0112 and mycological cure rate: 81.88% vs 66.67%; difference (95% CI): 15.21% (5.76, 24.66); *P* = 0.0019] (Fig. 2c; Table S3). Consistent results were observed in the subpopulation of subjects with positive *C. albicans* [therapeutic cure rate: 60.94% vs 45.45%; difference (95% CI): 15.48% (3.23, 27.74); *P* = 0.0144 and mycological cure rate: 92.19% vs 79.34%; difference (95% CI): 12.85% (4.27, 21.43); *P* = 0.0036] (Fig. 2d; Table S3). However, the clinical cure rate at D14 was similar between the two groups in the overall mITT population (56.88% in the oteseconazole group vs 50.31% in the fluconazole group, *P* = 0.2401) and in subjects with positive *C. albicans* (63.28% in the oteseconazole group vs 53.72% in the fluconazole group, *P* = 0.1257) (Table S3).

Antifungal rescue medication usage was reported in 3.75% of subjects receiving oteseconazole versus 14.47% of subjects receiving fluconazole, and such difference was statistically significant (*P* = 0.0009).

In the oteseconazole group, one subject missed one capsule of oteseconazole on D1 and another subject missed one capsule of fluconazole matching placebo on D4, and all the other subjects administered treatments per the protocol, indicating a high compliance. There were 51.3% and 42.9% of subjects in the oteseconazole group and the fluconazole group, respectively, reporting at least one treatment-emergent adverse event (TEAE), among whom 21.9% and 19.9% of subjects experienced at least one treatment-related adverse event (TRAE), respectively. All TEAEs were mild or moderate in severity. In the oteseconazole group, the most commonly reported TEAEs by PT were urinary tract infection (8.1%), bacterial vulvovaginitis (4.4%), dizziness (3.8%), and headache (3.8%); while in the fluconazole group, the most commonly reported TEAEs by PT were bacterial vulvovaginitis (7.5%), bacterial vaginosis (6.2%), urinary tract infection (4.3%), and nausea (3.1%) (Table 2). No subjects discontinued treatments or study due to TEAEs. No deaths occurred in this study. One subject in the fluconazole group reported a serious TEAE of threatened abortion, which was deemed as unrelated to the treatment by the investigator. During the study, there were no clinically significant changes in the vital signs, physical examination findings, ECGs, or laboratory parameters in either group.

**TABLE 2:** Treatment-emergent adverse events with an incidence >1% in either group by preferred term (*s*afety set)^*a*^

Preferred term	Oteseconazole (*N* = 160)	Fluconazole (*N* = 161)
Urinary tract infection	13 (8.1)	7 (4.3)
Bacterial vulvovaginitis	7 (4.4)	12 (7.5)
Dizziness	6 (3.8)	3 (1.9)
Headache	6 (3.8)	1 (0.6)
Nausea	5 (3.1)	5 (3.1)
Upper respiratory tract infection	5 (3.1)	4 (2.5)
Abdominal discomfort	4 (2.5)	1 (0.6)
Abdominal pain	3 (1.9)	1 (0.6)
Blood creatine phosphokinase increased	3 (1.9)	2 (1.2)
Diarrhoea	3 (1.9)	2 (1.2)
Lethargy	3 (1.9)	2 (1.2)
Abnormal uterine bleeding	2 (1.3)	0
Aspartate aminotransferase increased	2 (1.3)	1 (0.6)
Atrial escape rhythm	2 (1.3)	0
Bacterial vaginosis	2 (1.3)	10 (6.2)
Bilirubin conjugated increased	2 (1.3)	2 (1.2)
Blood cholesterol increased	2 (1.3)	1 (0.6)
Blood triglycerides increased	2 (1.3)	1 (0.6)
Blood uric acid increased	2 (1.3)	3 (1.9)
Dry mouth	2 (1.3)	0
Lipids increased	2 (1.3)	0
Menstrual disorder	2 (1.3)	0
Mycoplasma infection	2 (1.3)	1 (0.6)
Nasopharyngitis	2 (1.3)	0
Proteinuria	2 (1.3)	0
Rash	2 (1.3)	3 (1.9)
Urine ketone body present	2 (1.3)	0
White blood cells urine positive	2 (1.3)	0
Anaemia	1 (0.6)	4 (2.5)
Asthenia	1 (0.6)	3 (1.9)
Back pain	1 (0.6)	2 (1.2)
White blood cell count decreased	1 (0.6)	2 (1.2)
Abdominal pain upper	0	2 (1.2)
Decreased appetite	0	2 (1.2)
Neutrophil count decreased	0	2 (1.2)
QRS axis abnormal	0	2 (1.2)

^*a*^ Data are *n* (%). Abbreviations: *N*, number of subjects in the safety set.

## DISCUSSION

Primarily based on the global pivotal phase 3 studies of VIOLET (NCT03562156 and NCT03561701) and ultraVIOLET (NCT03840616), oteseconazole (Vivjoa) was approved by the Food and Drug Administration (FDA) to reduce the incidence of RVVC in April 2022. This randomized, double-blinded, positive-controlled, phase 3 study was conducted to evaluate the efficacy and safety of oteseconazole versus fluconazole in treating severe VVC in Chinese women.

After adequate discussion with and agreed upon by the investigators and health authority, we used a locally standardized VSS scoring system for screening and efficacy evaluations according to the current VVC diagnosis and treatment guidance in China (17). This scoring system is modified based on the global standard and is rendered to better suit the Chinese women suffering from VVC and reflect their conditions more accurately. The decision of choosing the time point of Day 28 to assess the efficacy primarily was in accordance with the FDA guidance on drug development for VVC treatments issued in 2019 (20), in which the primary endpoint for the efficacy evaluations of systemic drugs with long half-time was recommended to be assessed at D21 to D30.

The results of MICs for clinical isolates of *Candida* species and *in vitro* susceptibility test favored oteseconazole over fluconazole. At D28, oteseconazole demonstrated statistically significant and clinically meaningful superiority over fluconazole (66.88% vs 45.91%; *P* = 0.0002) in the primary endpoint of therapeutic cure rate, and consistent results were observed in the secondary endpoints of clinical cure and mycological cure rates at D28. In line with the reported epidemiology in VVC, *C. albicans* was the causative agent for the majority of subjects (78.1%) in this study (1). As expected, the results of therapeutic cure, clinical cure, and mycological cure rates at D28 in subjects tested positive for *C. albicans* were consistent with those in the overall mITT population. In addition to potent antifungal clinical activity in the treatment of VVC, oteseconazole treatment was potentially associated with reduced VVC recurrence and sustained treatment effect (13). In a phase 2 study in VVC subjects, subjects in the oteseconazole group showed no evidence of mycological recurrence whereas 28.6% and 46.1% of subjects in the fluconazole group had mycological recurrence at D84 and D168, respectively (13).

Oteseconazole was generally tolerated in subjects with severe VVC. The incidence of TEAEs was similar between the oteseconazole and the fluconazole groups. In the oteseconazole group, the most commonly reported TEAEs were urinary tract infection and bacterial vulvovaginitis, which were expected symptoms in subjects with severe VVC. Other commonly reported TEAEs in the oteseconazole group were mainly in the infection and infestations, gastrointestinal disorders, nervous system disorders, and system organ classes, which were also typical events of azole antifungal agents. In the fluconazole group, the most frequently observed TEAEs were bacterial vulvovaginitis, bacterial vaginosis, and urinary tract infection, which were similar to that reported in the previous study (15).

The potential pregnancy risk in the use of azole agents is an important concern for patients with VVC. The FDA has alerted the public that the use of chronic, high doses of fluconazole during the first trimester of pregnancy may be associated with birth defects (21). Based on the previous rat studies, oteseconazole is contraindicated in females of reproductive potential and in pregnant or lactating women (22). In the present study, nine subjects (two in the oteseconazole group and seven in the fluconazole group) reported pregnancy during the study, all chose elective termination. No treatment-related pregnancy risks were reported in this study or previous studies. At the time being, there are limited data on pregnant women who were exposed to oteseconazole during clinical trials, and these data are insufficient to exclude potential risks to human infants. Further studies are warranted to investigate the impact of oteseconazole on reproductivity and fertility and inform the optimal usage and relative precautions for women of reproductive potential.

This study had several limitations. Firstly, we did not perform long-term follow-up to evaluate the effect of oteseconazole on reducing VVC recurrence. However, in the three completed phase 3 studies (NCT03562156, NCT03561701, and NCT03840616) where subjects with RVVC were treated and followed up for more than 1 year, oteseconazole significantly reduced VVC recurrence rate. In addition, long-term follow-up did not result in any increase in toxicity, with no serious TRAEs or deaths reported. There were only few severe TEAEs leading to treatment discontinuations observed (15, 22, 23). Another limitation was that this study did not assess the antifungal effect of oteseconazole and fluconazole against non-*C. albicans* species due to the very small number of isolates. However, it is worth noting that preclinical studies of oteseconazole showed potent antifungal activity against non-*C. albicans* species both *in vitro* and *in vivo* (11, 12). Nonetheless, additional clinical data demonstrating such efficacy would be very insightful, especially considering that infections caused by non-*C. albicans* species are on the rise (24, 25). In the future, a global, multicenter trial to investigate the efficacy and safety of oteseconazole among a diverse population of patients presenting with various *Candida* species from diverse ethnic backgrounds is substantial.

In conclusion, this phase 3 study demonstrated statistically significant and clinically meaningful superiority of oteseconazole over fluconazole in the treatment of severe VVC. Additionally, oteseconazole was generally tolerated. The totality of evidence suggested that oteseconazole could serve as a treatment option in women suffering from severe VVC, with improved efficacy and a desirable safety profile.

## Data Availability

All the data underlying the findings of this manuscript are available from the corresponding author. Qualified scientific and medical researchers may request access to the data by sending an email to the corresponding author (qinping_liao@163.com); the corresponding author and sponsor will grant access to the data if the request is deemed appropriate.

## SUPPLEMENTAL MATERIAL

Tables S1 to S3 aac.00778-23-s0001.docx Susceptibility analysis and summary of efficacy results.

ASM does not own the copyrights to Supplemental Material that may be linked to, or accessed through, an article. The authors have granted ASM a non-exclusive, world-wide license to publish the Supplemental Material files. Please contact the corresponding author directly for reuse.
