# Supplementary material for: Oteseconazole versus fluconazole for the treatment of severe vulvovaginal candidiasis: a multicenter, randomized, double-blinded, phase 3 trial
Source: Antimicrob Agents Chemother. 2023 Dec 14;68(1):e00778-23. doi: 10.1128/aac.00778-23 (PMC10869335; doi:10.1128/aac.00778-23)
Supplement: Tables S1 to S3 — Susceptibility analysis and summary of efficacy results. [file aac.00778-23-s0001.docx]

**Supplementary**

Table of Contents

[Table S1 Susceptibility analyses of clinical isolated strains at baseline (mITT) 2](#_Toc142305114)

[Table S2 Summary of efficacy results at D28 visit (mITT) 4](#_Toc142305115)

[Table S3 Summary of efficacy results at D14 visit (mITT) 6](#_Toc142305116)

## Table S1 Susceptibility analyses of clinical isolated strains at baseline (mITT)

| **Fungal Species** |  | **Oteseconazole group**  **(N = 160)** | **Fluconazole group**  **(N = 159)** |
| --- | --- | --- | --- |
| *Candida albicans* | No. | 249 | 249 |
|  | Mean (SD) | 0.298 (1.011) | 3.589 (16.926) |
|  | Range | 0.032-8 | 0.064-256 |
|  | MIC_50_ | 0.125 | 1 |
|  | MIC_90_ | 0.25 | 4 |
| *Candida glabrata* | No. | 49 | 49 |
|  | Mean (SD) | 1.495 (1.851) | 23.020 (60.328) |
|  | Range | 0.125-8 | 1-256 |
|  | MIC_50_ | 1 | 8 |
|  | MIC_90_ | 4 | 16 |
| *Candida tropicalis* | No. | 8 | 8 |
|  | Mean (SD) | 2.083 (3.653) | 64.297 (118.322) |
|  | Range | 0.032-8 | 0.125-256 |
|  | MIC_50_ | 0.125 | 0.5 |
|  | MIC_90_ | 8 | 256 |
| *Candida krusei* | No. | 3 | 3 |
|  | Mean (SD) | 0.5 (0) | 9.333 (6.110) |
|  | Range | 0.5-0.5 | 4-16 |
|  | MIC_50_ | 0.5 | 8 |
|  | MIC_90_ | 0.5 | 16 |
| *Candida spherical* | No. | 4 | 4 |
|  | Mean (SD) | 0.032 (0) | 0.438 (0.125) |
|  | Range | 0.032-0.032 | 0.25-0.5 |
|  | MIC_50_ | 0.032 | 0.5 |
|  | MIC_90_ | 0.032 | 0.5 |
| *Candida parapsilosis* | No. | 3 | 3 |
|  | Mean (SD) | 0.032 (0) | 0.1667 (0.072) |
|  | Range | 0.032-0.032 | 0.125-0.25 |
|  | MIC_50_ | 0.032 | 0.125 |
|  | MIC_90_ | 0.032 | 0.25 |
| *Kodamaea ohmeri* | No. | 1 | 1 |
|  | Mean | 0.064 | 1 |
| *Candida dubliniensis* | No. | 1 | 1 |
|  | Mean | 0.032 | 0.25 |
| *Saccharomyces cerevisiae* | No. | 1 | 1 |
|  | Mean | 0.25 | 4 |
| *Candida lusitaniae* | No. | 1 | 1 |
|  | Mean | 0.125 | 0.25 |

Abbreviations: MIC_50_, minimum inhibitory concentration to inhibit 50% of isolates; MIC_90_, minimum inhibitory concentration to inhibit 90% of isolates; mITT, modified intention-to-treat; No., number of subjects; SD, standard deviation.

## Table S2 Summary of efficacy results at D28 visit (mITT)

| **mITT population** | | | |
| --- | --- | --- | --- |
|  | **Oteseconazole**  **(N = 160)** | | **Fluconazole**  **(N = 159)** |
| **Therapeutic Cure** |  | |  |
| No. | 107 | | 73 |
| Therapeutic cure rate, % (95% CI) | 66.88 (59.01, 74.10) | | 45.91 (37.99, 53.99) |
| Rate difference, % (95% CI) | 20.96 (10.32, 31.60) | | |
| *P* value | 0.0002 | | |
| **Mycological Cure** |  | |  |
| No. | 132 | | 94 |
| Mycological cure rate, % (95% CI) | 82.50 (75.71, 88.05) | | 59.12 (51.05, 66.84) |
| Rate difference, % (95% CI) | 23.38 (13.73, 33.03) | | |
| *P* value | <0.0001 | | |
| **Clinical Cure** |  | |  |
| No. | 114 | | 89 |
| Clinical cure rate, % (95% CI) | 71.25 (63.57, 78.12) | | 55.97 (47.89, 63.83) |
| Rate difference, % (95% CI) | 15.28 (4.85, 25.70) | | |
| *P* value | 0.0046 | | |
| **mITT subpopulation with positive culture for *Candida albicans*** | | | |
|  | **Oteseconazole**  **(N = 128)** | **Fluconazole**  **(N = 121)** | |
| **Therapeutic Cure** |  |  | |
| No. | 98 | 68 | |
| Therapeutic cure rate, % (95% CI) | 76.56 (68.26, 83.59) | 56.20 (46.89, 65.20) | |
| Rate difference, % (95% CI) | 20.36 (8.87, 31.85) | | |
| *P* value | 0.0007 | | |
| **Mycological Cure** |  |  | |
| No. | 118 | 86 | |
| Mycological cure rate, % (95% CI) | 92.19 (86.10, 96.19) | 71.07 (76.58, 86.50) | |
| Rate difference, % (95% CI) | 21.11 (11.79, 30.43) | | |
| *P* value | <0.0001 | | |
| **Clinical Cure** |  |  | |
| No. | 100 | 75 | |
| Clinical cure rate, % (95% CI) | 78.13 (69.96, 84.95) | 61.98 (52.71, 70.65) | |
| Rate difference, % (95% CI) | 16.14 (4.91, 27.37) | | |
| *P* value | 0.0053 | | |

Abbreviations: CI, confidence interval; mITT, modified intention-to-treat; No., number of subjects; VVC, vulvovaginal candidiasis.

## Table S3 Summary of efficacy results at D14 visit (mITT)

| **mITT population** | | | |
| --- | --- | --- | --- |
|  | **Oteseconazole**  **(N = 160)** | | **Fluconazole**  **(N = 159)** |
| **Therapeutic Cure** |  | |  |
| No. | 84 | | 61 |
| Therapeutic cure rate, (%) (95% CI) | 52.50 (44.47, 60.44) | | 38.36 (30.77, 46.40) |
| Rate difference (95% CI) | 14.14 (3.32, 24.95) | | |
| *P* value | 0.0112 | | |
| **Mycological Cure** |  | |  |
| No. | 131 | | 106 |
| Mycological cure rate, (%) (95% CI) | 81.88 (75.02, 87.51) | | 66.67 (58.77, 73.93) |
| Rate difference, (95% CI) | 15.21(5.76, 24.66) | | |
| *P* value | 0.0019 | | |
| **Clinical Cure** |  | |  |
| No. | 91 | | 80 |
| Clinical cure rate, (%) (95% CI) | 56.88 (48.82-64.67) | | 50.31 (42.29-58.33) |
| Rate difference, (95% CI) | 6.56 (-4.36, 17.48) | | |
| *P* value | 0.2401 | | |
| **mITT subpopulation with positive culture for *Candida albicans*** | | | |
|  | **Oteseconazole**  **(N = 128)** | **Fluconazole**  **(N = 121)** | |
| **Therapeutic Cure** |  |  | |
| No. | 78 | 55 | |
| Therapeutic cure rate, (%) (95% CI) | 60.94 (51.92, 69.44) | 45.45 (36.38, 54.76) | |
| Rate difference (95% CI) | 15.48 (3.23, 27.74) | | |
| *P* value | 0.0144 | | |
| **Mycological Cure** |  |  | |
| No. | 118 | 96 | |
| Mycological cure rate, (%) (95% CI) | 92.19 (86.10, 96.19) | 79.34 (71.03, 86.16) | |
| Rate difference, (95% CI) | 12.85 (4.27, 21.43) | | |
| *P* value | 0.0036 | | |
| **Clinical Cure** |  |  | |
| No. | 81 | 65 | |
| Clinical cure rate, (%) (95% CI) | 63.28 (54.31, 71.62) | 53.72 (44.43, 62.83) | |
| Rate difference, (95% CI) | 9.56 (-2.63, 21.76) | | |
| *P* value | 0.1257 | | |

Abbreviations: CI, confidence interval; mITT, modified intention-to-treat; No., number of subjects; VVC, vulvovaginal candidiasis.
